# Supplementary figures and images for: Fibroblast-expressed LRRC15 is a receptor for SARS-CoV-2 spike and controls antiviral and antifibrotic transcriptional programs
Source: PLoS Biol. 2023 Feb 9;21(2):e3001967. doi: 10.1371/journal.pbio.3001967 (PMC9910744; doi:10.1371/journal.pbio.3001967)

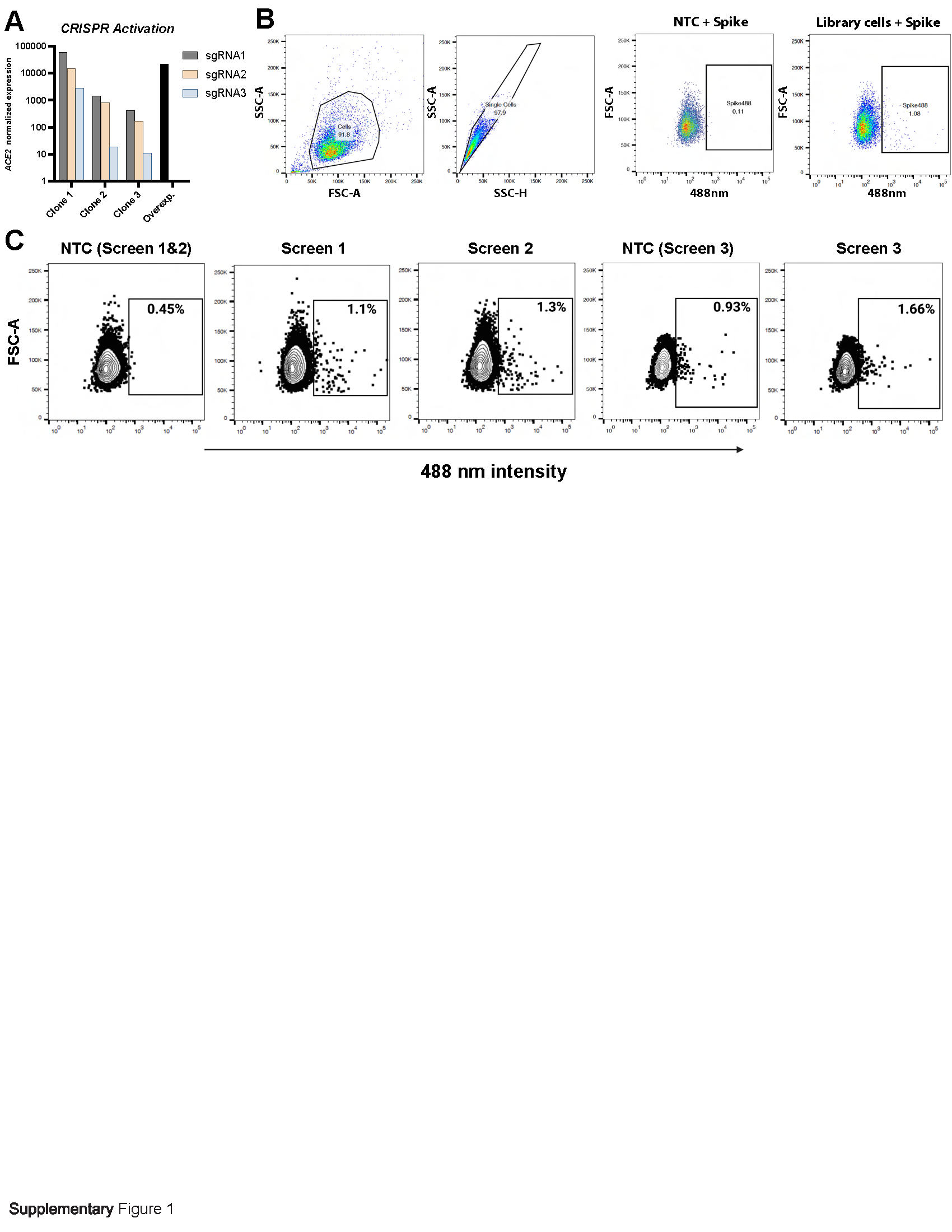

Supplement: S1 Fig — (A) RT-qPCR of ACE2 expression in SAM clonal cell lines transduced with ACE2 sgRNAs or with HEK293T-ACE2 cells. Results calculated using ΔΔCT method and normalized to NTC sgRNA-transduced HEK293T-CRISPRa cells. (B) FACS gating strategy. Cells were first gated by forward (FSC) and side scatter (SSC) before filtering for singlets. Spike488 fluorescence was gated by comparison with NTC sgRNA transduced cells. Similar strategy was applied to all flow cytometry experiments. (C) FACS results for 3 whole-genome CRISPRa screens with NTC sgRNA-transduced cells as negative controls. For screen 1, cells were incubated with Alexa Fluor 488-conjugated SARS-CoV-2 HexaPro spike (Addgene #154754) and selected on puromycin for 3 days. For screen 2, cells were incubated with Alexa Fluor 488-conjugated SARS-CoV-2 Spike glycoprotein (residues 1–1208, complete ectodomain; gift from Dr. Florian Krammer) and selected on puromycin for 3 days. For screen 3, cells were incubated with Alexa Fluor 488-conjugated SARS-CoV-2 HexaPro spike (Addgene #15474) and selected on puromycin for 8 days. The data underlying all panels in this figure can be found in DOI: 10.5281/zenodo.7416876. (TIFF) [file pbio.3001967.s001.tiff]

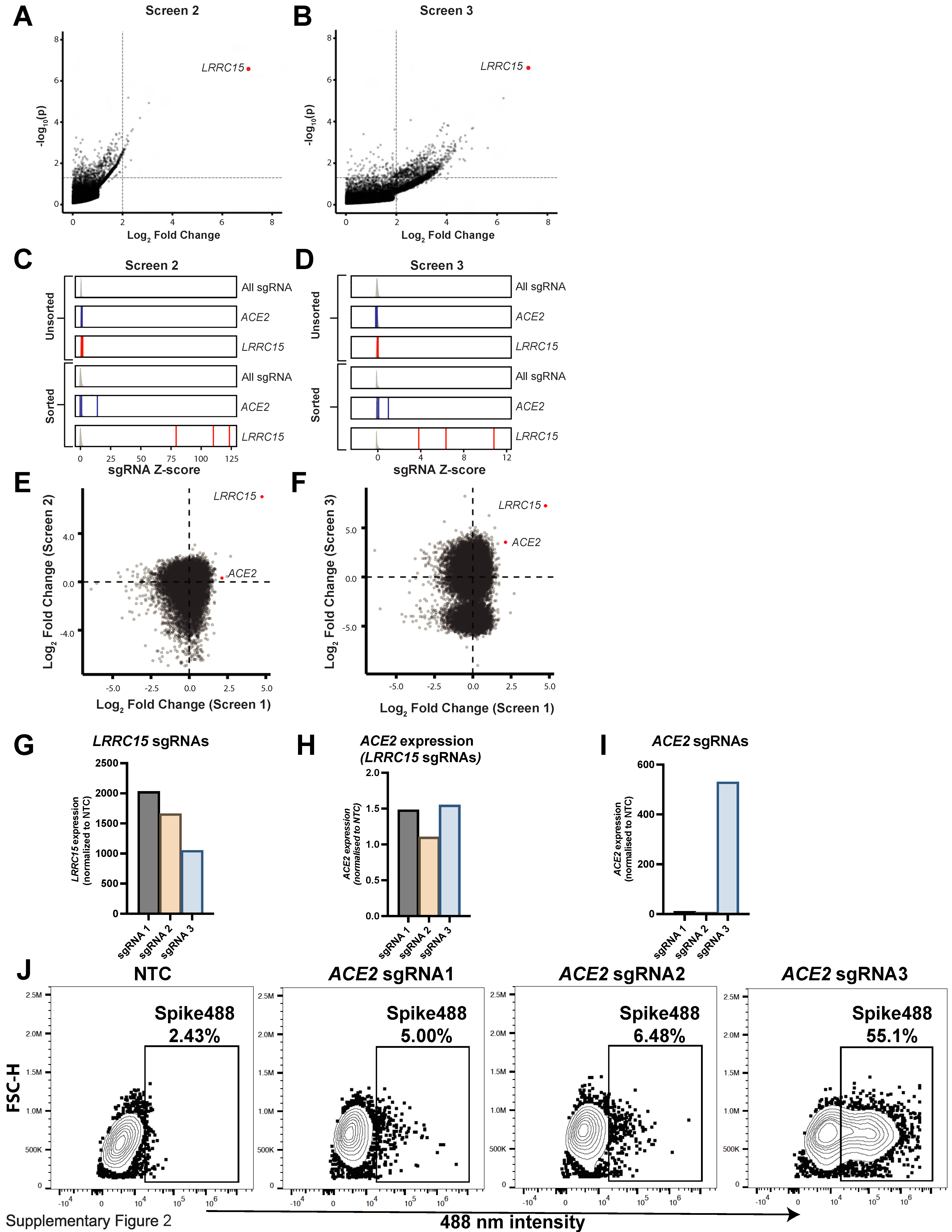

Supplement: S2 Fig — (A and B) Gene enrichment analysis of screens 2 (A) and 3 (B) performed using MAGeCK. Horizontal dotted line indicates p-value = 0.05. Vertical dotted line indicates log2 fold changes of 2. P-values and LFCs for all genes in screens 2 and 3 are reported in S1 Table. (C-D) Density plot of Z-score (gray) for all sgRNA in (C) screen 2 and (D) screen 3. Blue vertical lines indicate Z-score for ACE2 sgRNAs. Red vertical lines indicate Z-score for LRRC15 sgRNAs. Z-scores calculated as described in methods. (E) Log2 fold changes of all genes in screen 1 vs. log2 fold changes of all genes in screen 2. (F) Log2 fold changes of all genes in screen 1 vs. log2 fold changes of all genes in screen 3. (G) LRRC15 expression of cells in Fig 2E quantified via RT-qPCR. (H) ACE2 expression was not increased in LRRC15 sgRNA transduced cells (quantified via RT-qPCR). (I) The 3 sgRNAs for ACE2 from the Calabrese library used in our screens were transduced into HEK293T-CRISPRa cells, and ACE2 expression was confirmed via qPCR. Only sgRNA3 induced upregulation in ACE2 expression. (J) Transduced cells in (I) were incubated with Spike647 and analyzed via flow cytometry. Only ACE2 sgRNA3 cells showed a significant increase in Spike647 binding. The data underlying all panels in this figure can be found in DOI: 10.5281/zenodo.7416876. (TIFF) [file pbio.3001967.s002.tiff]

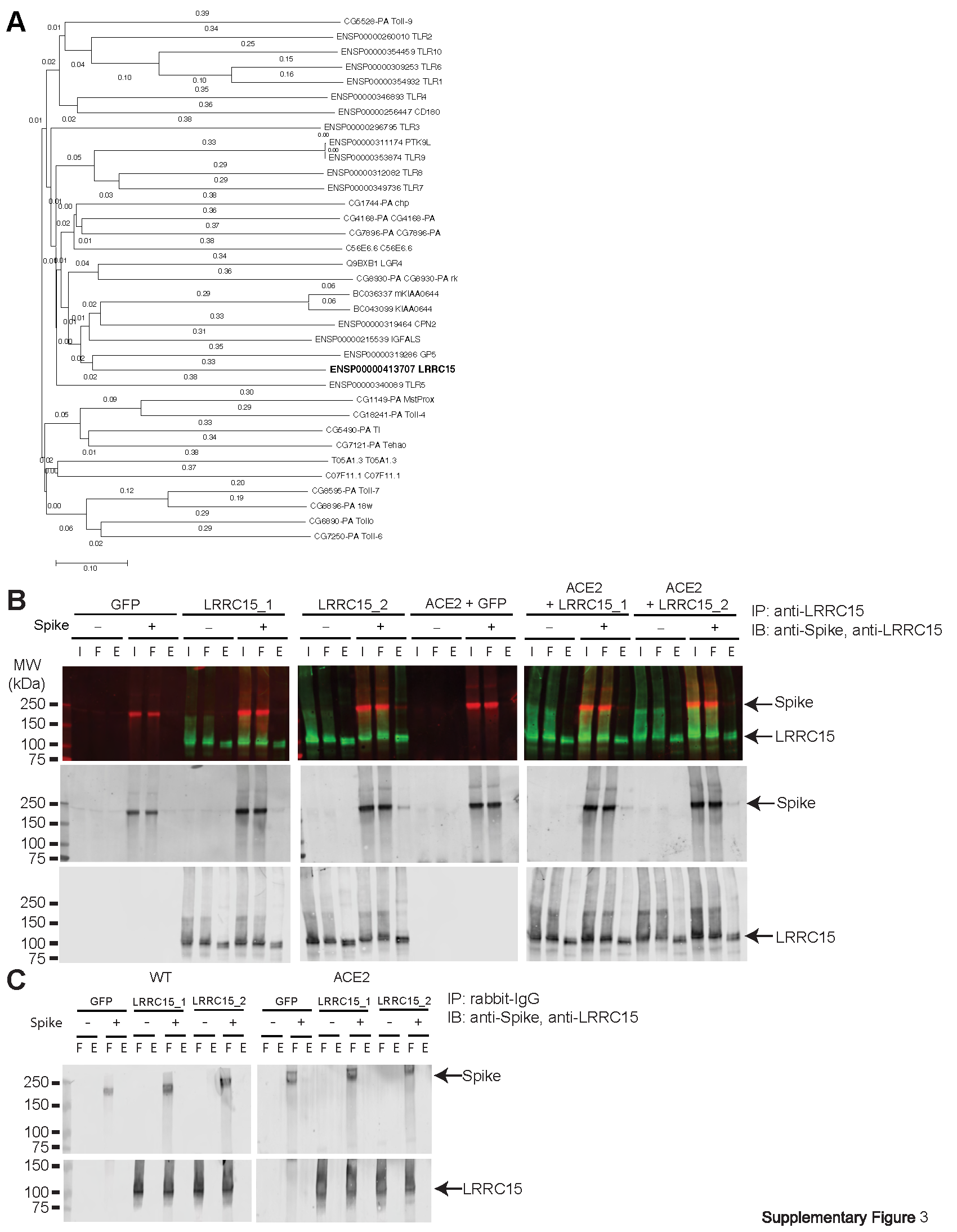

Supplement: S3 Fig — (A) Full phylogenetic tree of LRR-Tollkin family of proteins (includes fly and worm orthologs). (B) Co-immunoprecipitation of spike was observed in LRRC15-GFP (transcripts 1 and 2) and ACE2 expressing cells but not in control GFP cells. I = input, FT = flow-through, E = elute. (C) Control rabbit IgG did not immunoprecipitate LRRC15 or spike. The data underlying all panels in this figure can be found in DOI: 10.5281/zenodo.7416876. (TIFF) [file pbio.3001967.s003.tiff]

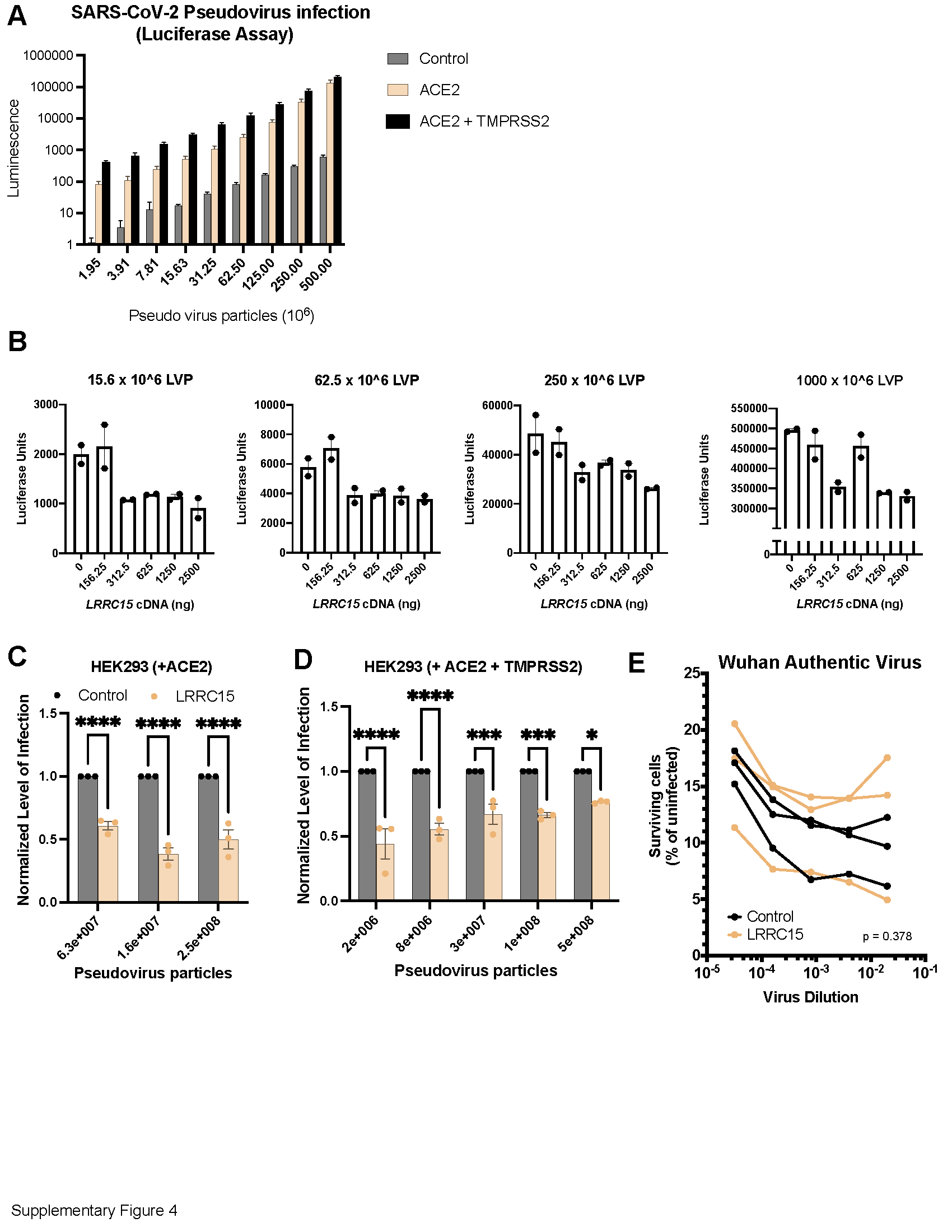

Supplement: S4 Fig — (A) SARS-CoV-2 pseudovirus carrying a firefly luciferase cassette was applied to HEK293T, HEK293T-ACE2, and HEK293T-ACE2-TMPRSS2 cells for 24 h before luminescence quantification. HEK293T cells were relatively resistant to infection, while HEK293T-ACE2 and HEK293T-ACE2-TMPRSS2 expressing cells were infectable. N = 3 for each condition. (B) Pseudovirus added to ACE2-expressing cells in the context of LRRC15. Titration of 15 × 106, 62.5 × 106, 250 × 106, and 1,000 × 10^6^ lentiviral particles in HEK293T-ACE2 cells transfected with 0, 156.25, 312.5, 625, 1,250, and 2,500 ng of Myc-DDK-tagged LRRC15 plasmid DNA. N = 2 for each condition. (C and D) Luciferase assay for quantification of SARS-CoV-2 pseudovirus infection in (C) HEK293T-ACE2 and (D) HEK293T-ACE2-TMPRSS2 (N = 3). Cells were transfected with plasmid encoding Myc-DDK-tagged LRRC15 transcript 1 or empty vector as a control. Luminescence for LRRC15 cells were normalized to control cells. Significance was determined by two-way ANOVA, Sidak multiple comparison test; ****p < 0.0001, ***p < 0.001, **p < 0.01, *p < 0.05. (E) Quantification of cell survival after incubation with authentic SARS-CoV-2 virus in HEK293T-ACE2-TMPRSS2 cells transfected with plasmid encoding Myc-DDK-tagged LRRC15 transcript 1 or Myc-DDK only (N = 3). Significance was determined by two-way ANOVA, *p < 0.05. The data underlying all panels in this figure can be found in DOI: 10.5281/zenodo.7416876. (TIFF) [file pbio.3001967.s004.tiff]

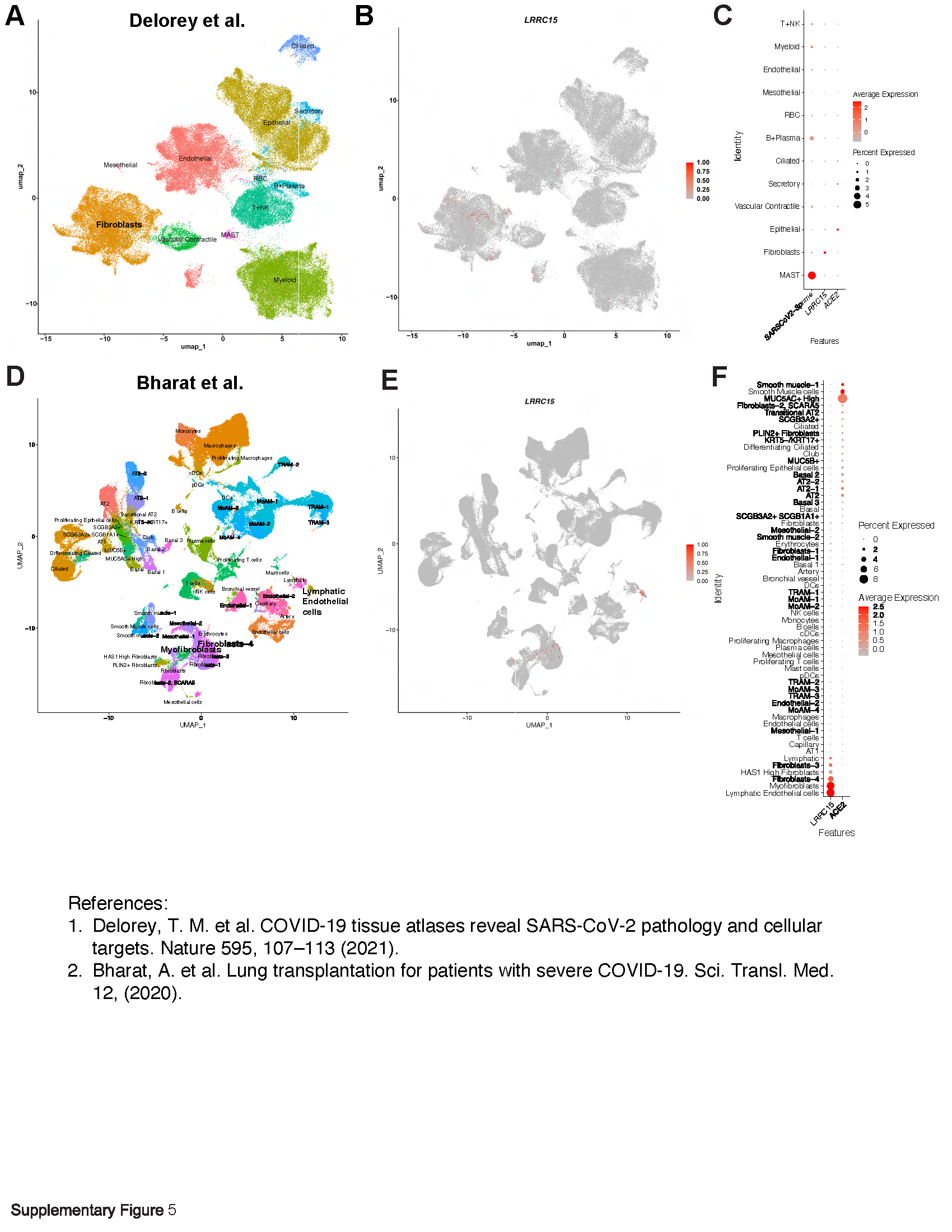

Supplement: S5 Fig — (A) UMAP plot of lung single-nucleus RNA seq dataset (Delorey and colleagues). (B) Feature plot and (C) dotplot shows LRRC15 is expressed in Delorey and colleagues fibroblasts. (D) UMAP plot of lung single-nucleus RNA seq dataset (Bharat and colleagues). (E) Feature plot and (F) dotplot shows LRRC15 is expressed in Bharat and colleagues lymphatic endothelial cells and various populations of fibroblasts. The data underlying all panels in this figure can be found in DOI: 10.5281/zenodo.7416876. (TIFF) [file pbio.3001967.s005.tiff]

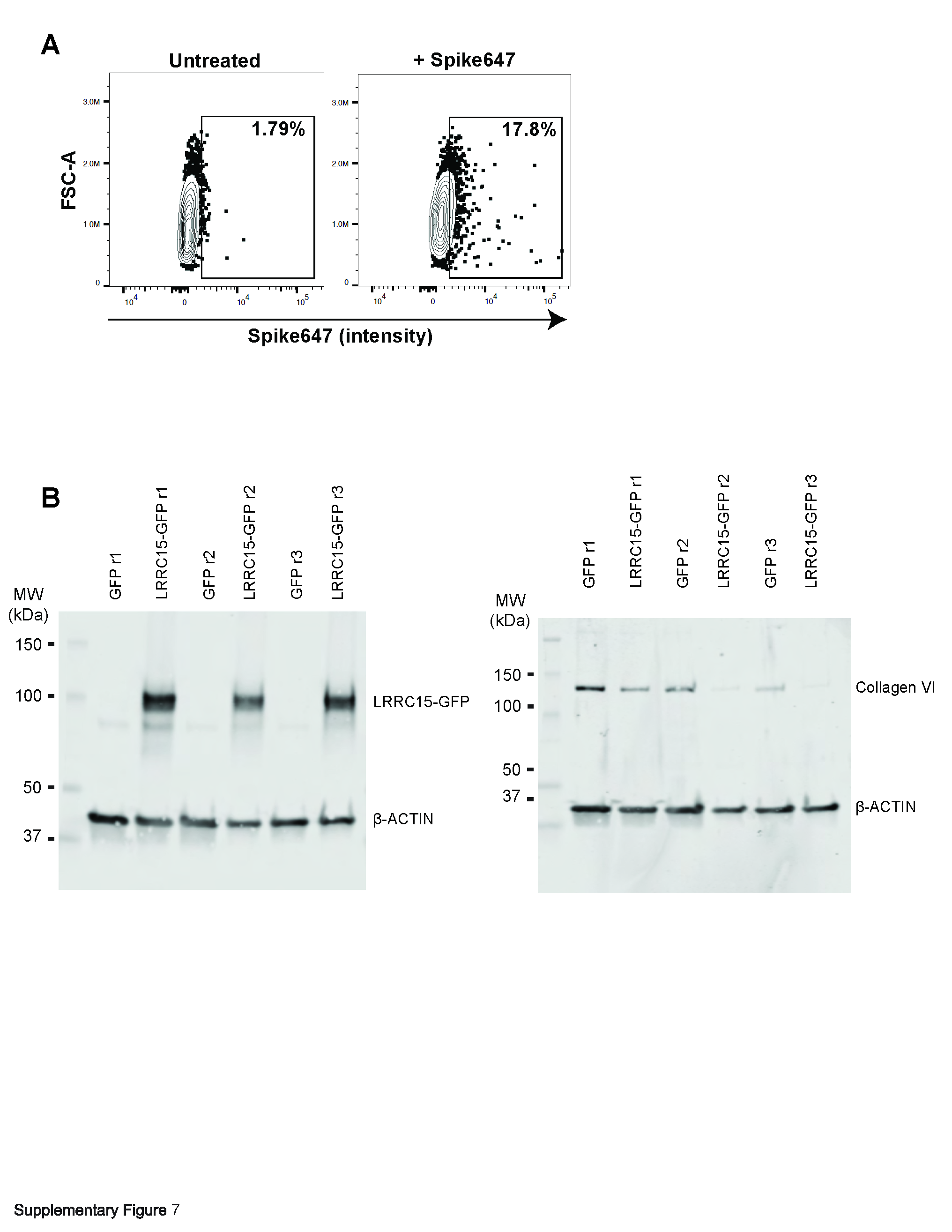

Supplement: S7 Fig — (A) Representative flow cytometry analysis of IMR90 fibroblasts incubated with Spike647 show cells have intrinsic spike-binding activity (N = 2). (B) Full images for LRRC15 and Collagen VI western blots. LRRC15 overexpression in fibroblasts results in decreased Collagen VI protein expression. The data underlying all panels in this figure can be found in DOI: 10.5281/zenodo.7416876. (TIFF) [file pbio.3001967.s007.tiff]
