# Supplementary material for: Fibroblast-expressed LRRC15 is a receptor for SARS-CoV-2 spike and controls antiviral and antifibrotic transcriptional programs
Source: PLoS Biol. 2023 Feb 9;21(2):e3001967. doi: 10.1371/journal.pbio.3001967 (PMC9910744; doi:10.1371/journal.pbio.3001967)
Supplement: S3 Table — List of primers used for next-generation sequencing of gDNA extracted from pooled CRISPR activation screen samples. Primers were adapted from Sanson and colleagues [5]. (DOCX) [file pbio.3001967.s010.docx]

# Supplementary Table 3: Primers for Next-Generation Sequencing of CRISPRa sgRNA from transduced cell gDNA

- Primer sequences adapted from Sanson et al[1]
- Primer regions:
  - P5/P7 flowcell attachment sequence
  - Illumina sequencing primer
  - Vector primer binding sequence
  - Stagger region / Barcode region

| **P5 Primers** | |
| --- | --- |
| **Stagger** | **Sequence** |
| **0** | AATGATACGGCGACCACCGAGATCTACACTCTTTCCCTACACGACGCTCTTCCGATCTCTTGTGGAAAGGACGAAACACC |
| **1** | AATGATACGGCGACCACCGAGATCTACACTCTTTCCCTACACGACGCTCTTCCGATCTGTCTTGTGGAAAGGACGAAACACC |
| **2** | AATGATACGGCGACCACCGAGATCTACACTCTTTCCCTACACGACGCTCTTCCGATCTAGTCTTGTGGAAAGGACGAAACACC |
| **3** | AATGATACGGCGACCACCGAGATCTACACTCTTTCCCTACACGACGCTCTTCCGATCTGCCTCTTGTGGAAAGGACGAAACACC |
| **4** | AATGATACGGCGACCACCGAGATCTACACTCTTTCCCTACACGACGCTCTTCCGATCTACAATCTTGTGGAAAGGACGAAACACC |
| **5** | AATGATACGGCGACCACCGAGATCTACACTCTTTCCCTACACGACGCTCTTCCGATCTTAGAGTCTTGTGGAAAGGACGAAACACC |
| **6** | AATGATACGGCGACCACCGAGATCTACACTCTTTCCCTACACGACGCTCTTCCGATCTCAGCAATCTTGTGGAAAGGACGAAACACC |
| **7** | AATGATACGGCGACCACCGAGATCTACACTCTTTCCCTACACGACGCTCTTCCGATCTTGAGACATCTTGTGGAAAGGACGAAACACC |

| **P7 Primers** | |
| --- | --- |
| **Index** | **Sequence** |
| 5 | CAAGCAGAAGACGGCATACGAGATCACGATGTGACTGGAGTTCAGACGTGTGCTCTTCCGATCTACCGACTCGGTGCCACTTTTTCAAG |
| 6 | CAAGCAGAAGACGGCATACGAGATCAGGCGGTGACTGGAGTTCAGACGTGTGCTCTTCCGATCTACCGACTCGGTGCCACTTTTTCAAG |
| 7 | CAAGCAGAAGACGGCATACGAGATTACAGCGTGACTGGAGTTCAGACGTGTGCTCTTCCGATCTACCGACTCGGTGCCACTTTTTCAAG |

**References:**

1. Sanson KR, Hanna RE, Hegde M, Donovan KF, Strand C, Sullender ME, et al. Optimized libraries for CRISPR-Cas9 genetic screens with multiple modalities. Nat Commun. 2018 Dec 21;9(1):5416.
