# Supplementary material for: Fibroblast-expressed LRRC15 is a receptor for SARS-CoV-2 spike and controls antiviral and antifibrotic transcriptional programs
Source: PLoS Biol. 2023 Feb 9;21(2):e3001967. doi: 10.1371/journal.pbio.3001967 (PMC9910744; doi:10.1371/journal.pbio.3001967)
Supplement: S4 Table — List of primers used for RT-qPCR. (DOCX) [file pbio.3001967.s011.docx]

# Supplementary Table 4: Primers for RT-qPCR

| **Primer** | **Sequence (5’ → 3’)** | **Source** |
| --- | --- | --- |
| *GAPDH* Fwd | ACAACTTTGGTATCGTGGAAGG | Primerbank[1] (Primerbank ID: 378404907c2) |
| *GAPDH* Rev | GCCATCACGCCACAGTTTC | Primerbank[1] (Primerbank ID: 378404907c2) |
| *ACE2* Fwd | GGACCCAGGAAATGTTCAGA | Jia et al., 2020[2] |
| *ACE2* Rev | GGCTGCAGAAAGTGACATGA | Jia et al., 2020[2] |
| *LRRC15* Fwd | GCCTTTGGACAAGGCTATGC | Wang et al., 2018[3] |
| *LRRC15* Rev | GAGCAGGTACACTCGCTAGG | Wang et al., 2018[3] |
| *COL1A1* Fwd | CAAGACGAAGACATCCCACCA | This paper |
| *COL1A1* Rev | AGTAGCACCATCATTTCCACGA | This paper |
| *COL1A2* Fwd | GTGGCAGTGATGGAAGTGTG | Yu et al., 2018[4] |
| *COL1A2* Rev | AGGACCAGCGTTACCAACAG | Yu et al., 2018[4] |
| *COL6A1* Fwd | GACCTCGGACCTGTTGGGTAC | Yuri et al., 2020[5] |
| *COL6A1* Rev | TACCCCATCTCCCCCTTCAC | Yuri et al., 2020[5] |
| *COL6A2* Fwd | CAGGAGGTCATCTCGCCG | Piao et al., 2021[6] |
| *COL6A2* Rev | GTTCTGCAGCTGGCTGATG | Piao et al., 2021[6] |
| *COL6A3* Fwd | CCTAACCACATATGTTAGTGGAGGT | Dankel et al., 2020[7] |
| *COL6A3* Rev | GAATGTCTCGCTTGCTCTCTG | Dankel et al., 2020[7] |
| *COL12A1* Fwd | AAAGGGGAAAGGAAATCAGC | Mohassel et al., 2019[8] |
| *COL12A1* Rev | TCACAGCATCTGTCTCTACTGGT | Mohassel et al., 2019[8] |
| *IFIT1* Fwd | GGAATACACAACCTACTAGCC | Li et al., 2019[9] |
| *IFIT1* Rev | CCAGGTCACCAGACTCCTCA | Li et al., 2019[9] |
| *IFIT3* Fwd | TGAGGAAGGGTGGACACAACTGAA | Li et al., 2019[9] |
| *IFIT3* Rev | AGGAGAATTCTGGGTTGTTGGGCT | Li et al., 2019[9] |
| *MX1* Fwd | AGGACCATCGGAATCTTGAC | Ortiz et al., 2020[10] |
| *MX1* Rev | TCAGGTGGAACACGAGGTTC | Ortiz et al., 2020[10] |
| *OAS1* Fwd | GCGCCCCACCAAGCTCAAGA | Li et al., 2019[9] |
| *OAS1* Rev | GCTCCCTCGCTCCCAAGCAT | Li et al., 2019[9] |
| *OAS2* Fwd | ACCCGAACAGTTCCCCCTGGT | Li et al., 2019[9] |
| *OAS2* Rev | ACAAGGGTACCATCGGAGTTGCC | Li et al., 2019[9] |

**References**

1. Spandidos A, Wang X, Wang H, Seed B. PrimerBank: a resource of human and mouse PCR primer pairs for gene expression detection and quantification. Nucleic Acids Res. 2010 Jan;38(Database issue):D792-9.

2. Jia HP, Look DC, Shi L, Hickey M, Pewe L, Netland J, et al. ACE2 receptor expression and severe acute respiratory syndrome coronavirus infection depend on differentiation of human airway epithelia. J Virol. 2005 Dec;79(23):14614–21.

3. Wang Y, Liu Y, Zhang M, Lv L, Zhang X, Zhang P, et al. LRRC15 promotes osteogenic differentiation of mesenchymal stem cells by modulating p65 cytoplasmic/nuclear translocation. Stem Cell Res Ther. 2018 Mar 9;9(1):65.

4. Yu Y, Liu D, Liu Z, Li S, Ge Y, Sun W, et al. The inhibitory effects of COL1A2 on colorectal cancer cell proliferation, migration, and invasion. J Cancer. 2018 Jul 30;9(16):2953–62.

5. Hou T, Tong C, Kazobinka G, Zhang W, Huang X, Huang Y, et al. Expression of COL6A1 predicts prognosis in cervical cancer patients. Am J Transl Res. 2016 Jun 15;8(6):2838–44.

6. Piao XM, Hwang B, Jeong P, Byun YJ, Kang HW, Seo SP, et al. Collagen type VI‑α1 and 2 repress the proliferation, migration and invasion of bladder cancer cells. Int J Oncol [Internet]. 2021 Jul;59(1). Available from: http://dx.doi.org/10.3892/ijo.2021.5217

7. Dankel SN, Grytten E, Bjune JI, Nielsen HJ, Dietrich A, Blüher M, et al. COL6A3 expression in adipose tissue cells is associated with levels of the homeobox transcription factor PRRX1. Sci Rep. 2020 Nov 19;10(1):20164.

8. Mohassel P, Liewluck T, Hu Y, Ezzo D, Ogata T, Saade D, et al. Dominant collagen XII mutations cause a distal myopathy. Ann Clin Transl Neurol. 2019 Oct;6(10):1980–8.

9. Li D, Swaminathan S. Human IFIT proteins inhibit lytic replication of KSHV: A new feed-forward loop in the innate immune system. PLoS Pathog. 2019 Feb;15(2):e1007609.

10. Ortiz E, Sanchis P, Bizzotto J, Lage-Vickers S, Labanca E, Navone N, et al. Myxovirus Resistance Protein 1 (MX1), a Novel HO-1 Interactor, Tilts the Balance of Endoplasmic Reticulum Stress towards Pro-Death Events in Prostate Cancer. Biomolecules [Internet]. 2020 Jul 6;10(7). Available from: http://dx.doi.org/10.3390/biom10071005
