# Supplementary material for: Fibroblast-expressed LRRC15 is a receptor for SARS-CoV-2 spike and controls antiviral and antifibrotic transcriptional programs
Source: PLoS Biol. 2023 Feb 9;21(2):e3001967. doi: 10.1371/journal.pbio.3001967 (PMC9910744; doi:10.1371/journal.pbio.3001967)

Raw blot image for Fig 3I, cropped images shown inset.

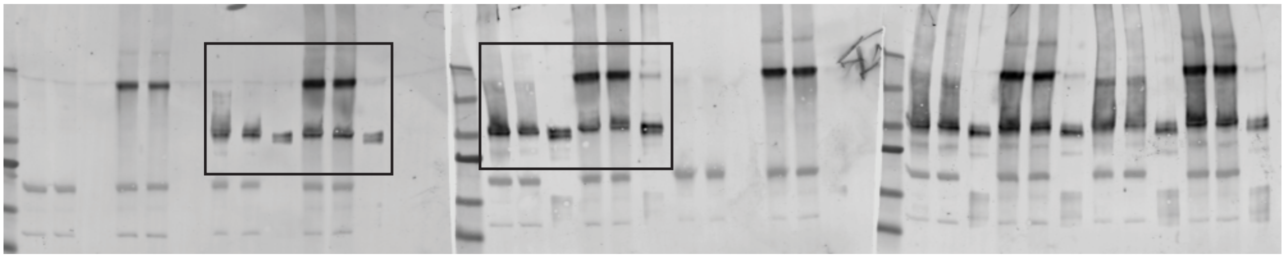

Raw blot images for S3B

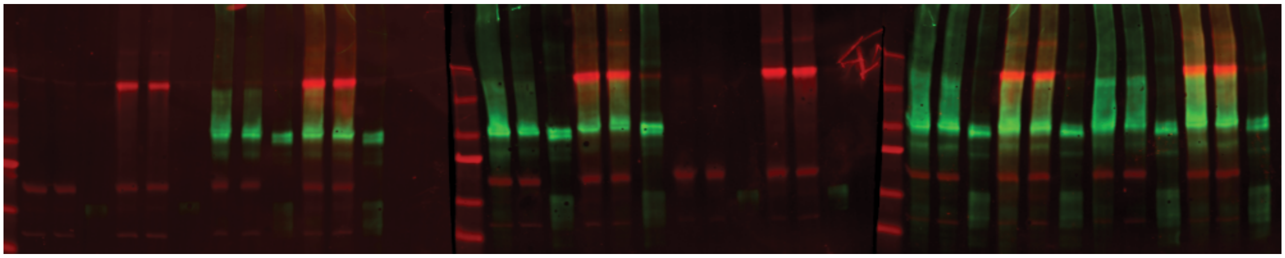

LRRC15 (green to black)

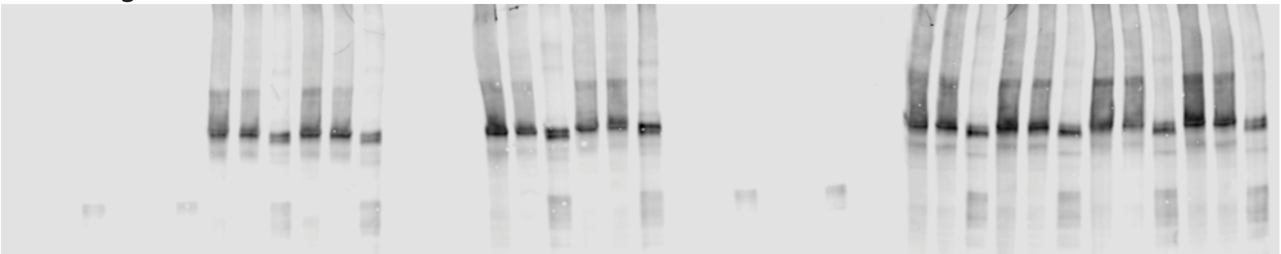

Spike (red to black)

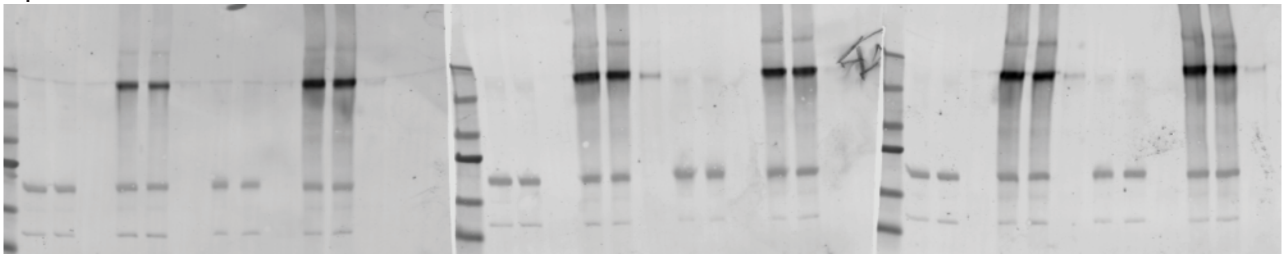

Raw blot images for S3C

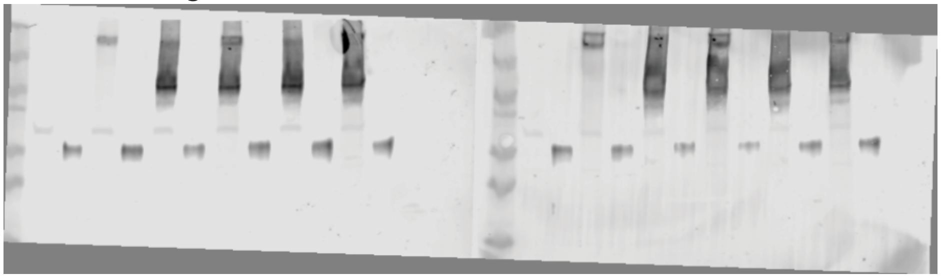

Full blot image for Collagen VI , Fig 6F. Top band Collagen, bottom band actin.
Shown in Supp Fig 7

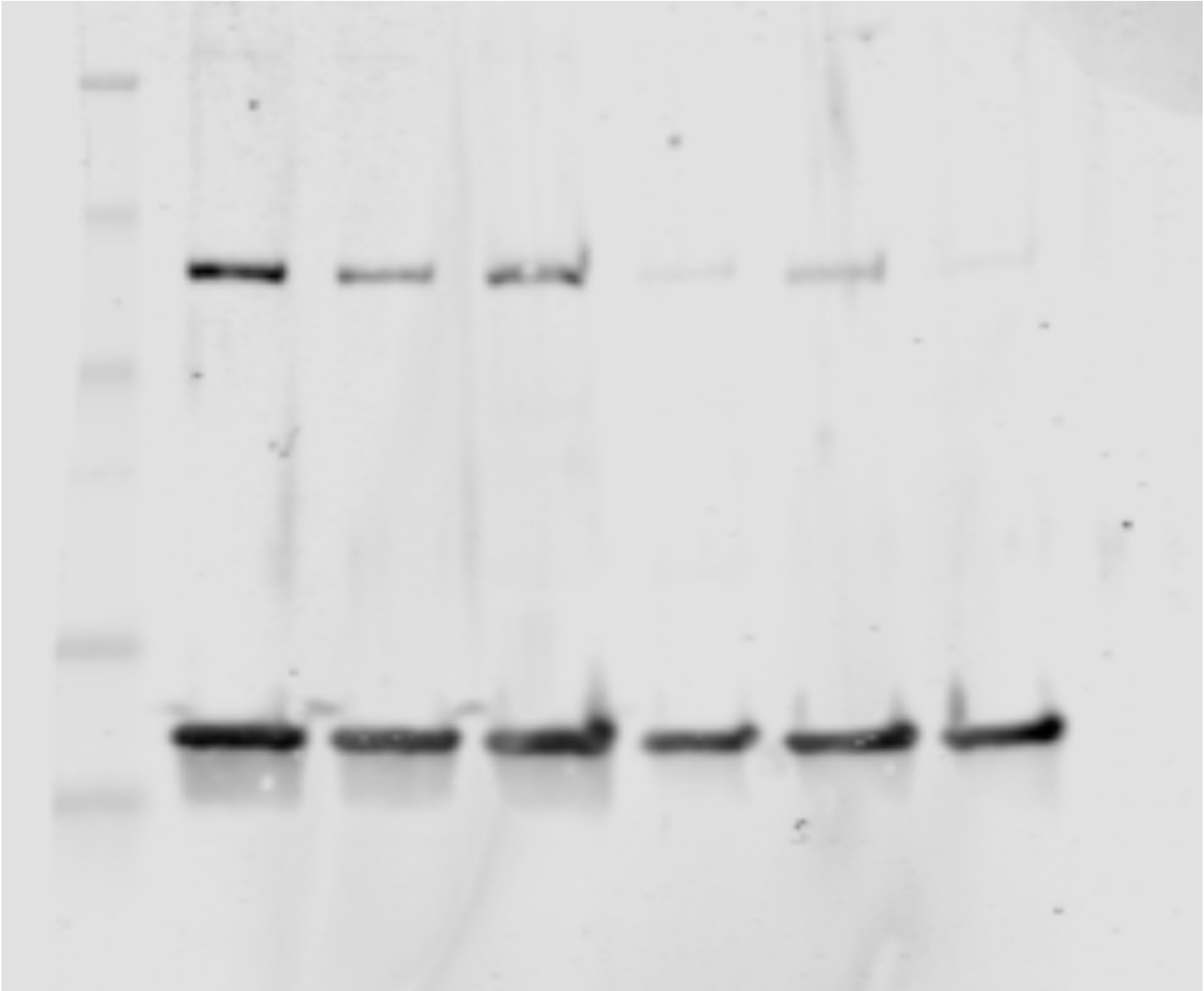

Full blot image for LRRC15, for Fig 6F. Top band LRRC15, bottom band actin.
Shown in Supp Fig 7

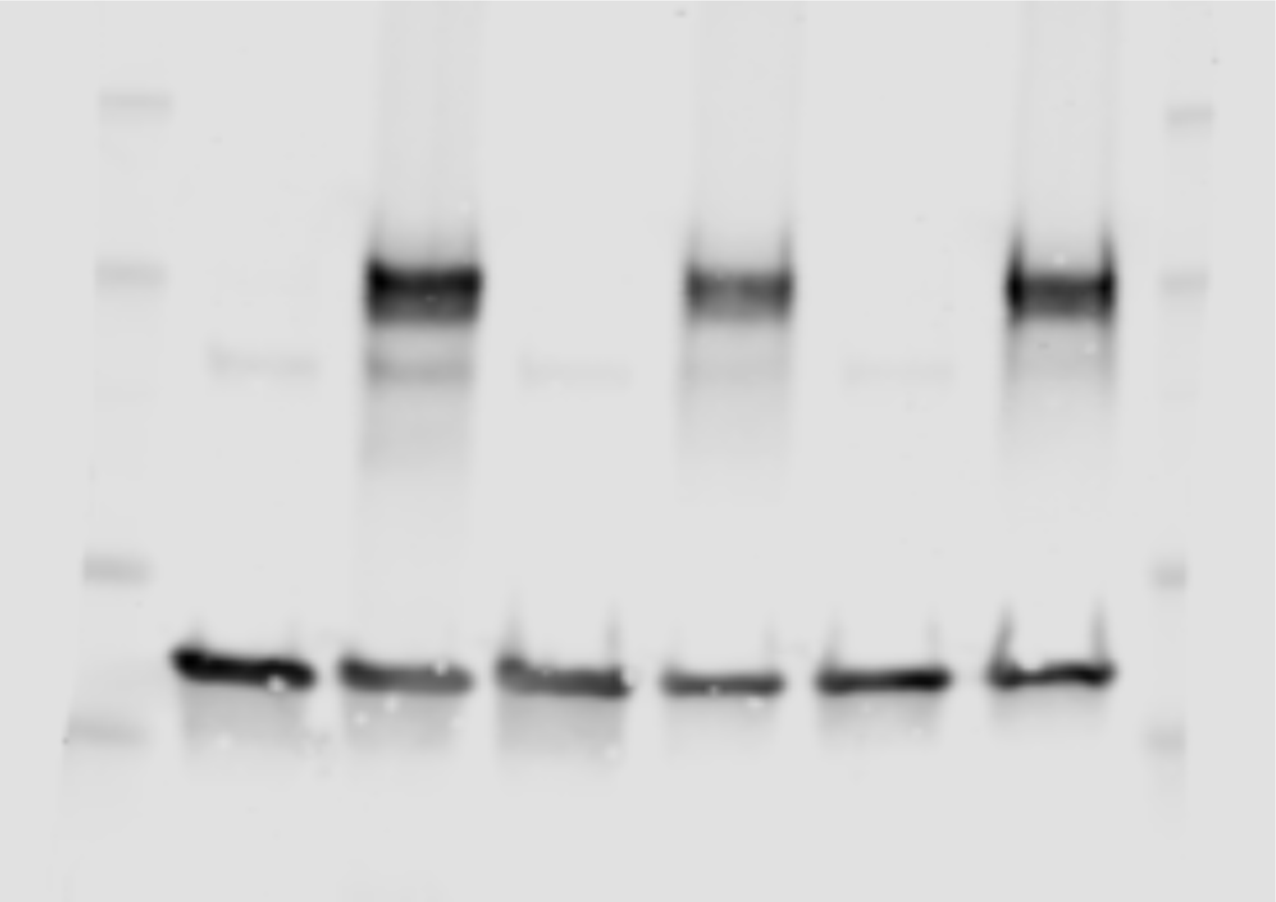

Supplement: S1 Raw images — (PDF) [file pbio.3001967.s014.pdf]
