# Supplementary material for: Fibroblast-expressed LRRC15 is a receptor for SARS-CoV-2 spike and controls antiviral and antifibrotic transcriptional programs
Source: PLoS Biol. 2023 Feb 9;21(2):e3001967. doi: 10.1371/journal.pbio.3001967 (PMC9910744; doi:10.1371/journal.pbio.3001967)
Supplement: Attachment — Submitted filename: renamed_16372.docx [file pbio.3001967.s015.docx]

**Point-by-point response to reviewer’s comments**

**Reviewer #1 (innate immunity, inflammation):**

*1) First and foremost I would strongly encourage the authors to change the conclusion which is prevalent throughout the paper. “Overall, we show LRRC15 is a master regulator of SARS-CoV-2 infection outcomes, physically linking SARS-CoV-2 to perturbed-state fibroblasts, collagen production and fibrosis associated with “long-haul” COVID.”*

*I think “master regulator” is somewhat exaggerated. I do not see evidence to suggest that LRRC15 mediates outcomes. If that receptor is indeed the master regulator it would be more important than ACE2 no?*

*Second, I see no data on long-haul COVID. I recommend toning down this conclusion.*

**Response**: To address this comment we have significantly revised the text and toned-down claims of LRRC15 acting as a “master regulator” of infection and also toned down its role in “long haul COVID”.

*2) The conclusion that LRRC15 reduces viral infection is interesting and needs to be explained based on location. Presumably these fibroblasts do not colocalize with epithelial cells that get infected ie., in the alveoli and bronchioles so the virus would see the epithelium first. While the LRRC15 may prevent dissemination into the interstitium of the lung, I fail to see how it would ever reduce infection of epithelium. This needs to be better explained.*

**Response**: To address this concern we have stained the lungs of COVID-19 patients with anti-collagen and anti-LRRC15 antibodies. Please see new figure 4 e-i and new supplementary figure 6. We find that LRRC15 is absent in non-COVID control lungs, but is highly upregulated in the human COVID lung, where LRRC15 protein was found to line the airways.

*3) The authors report a paper in bioarchives with very similar results. There is a second paper in bioarchives with similar results by Song et al., with identical conclusions. It too could be referenced but I agree the paper is timely.*

**Response**: The Song et al paper came out after we had submitted our manuscript for review, we have now added this bioarchives citation and also a citation for the accepted Song et al manuscript.

*4) The authors argue that the Delta variant has adapted to LRRC15 but could the authors speculate on a mechanism? It would not be SPIKE mutations so what other mutations are found in Delta versus D614G.*

**Response**: We have removed data and discussion on Delta and D614G and have focused our efforts on the original Wuhan variant.

*5) Long-COVID is different from the rapid fibrosis that can occur in patients that includes brain fog and other symptoms. I am not certain I would associate the long haul COVID with the increased fibrosis. I recommend consultation with physicians that may be able to help with this important issue.*

**Response**: We understand this concern and have toned down our claims about long haul COVID and instead focused on our data that LRRC15 can suppress expression of collagen.

**Reviewer #2 (viral immunity, SARS-CoV2):**

1) the conclusions that LRRC15 is a significant regulator of SARS-CoV-2 infection is not supported by the current data. Lack of data describing the mechanism by which LRRC15 expression regulates collagen production also dampens enthusiasm for the current manuscript.

**Response:** To address this concern, we have toned down our claims that LRRC15 is a “master regulator” of COVID-19 infection. To address mechanism, the primary mechanism of action for LRRC15 in antagonising SARS-CoV-2 infection is via direct interactions with the spike protein, and the two other manuscripts that found LRRC15 show a similar effect. Beyond this direct interaction with Spike, we have now expanded our understanding of how LRRC15 expression impacts the fibroblast, and found that ectopic expression of LRRC15 induces antiviral programs and suppresses collagen, which we confirm with qPCR and western blot, see new figure 6 d-f. We think this new knowledge can help the field better understand how this relatively uncharacterized molecule alters cellular physiology. We are interested in providing further mechanistic data on precisely how LRRC15 impacts fibroblast transcriptional programs, but this will take us years and we feel this is beyond the scope of this current study.

*2). The current data does not support LRRC15 as a master regulator of SARS-CoV-2 infection that sequesters virus from target cells. To play an important role in SARS-CoV-2 sequestration in vivo, the effect size (the degree to which this mechanism suppresses infection), relative positioning of LRRC15 fibroblasts to target cells, and timing post-infection are all important and inadequately addressed.*

**Response**. We have toned down our claims that LRRC15 is a master regulator of SARS-CoV-2 infection. Moreover, we now show that LRRC15 protein is upregulated in the lung of COVID-19 patients where it lines the airways. See new figure 4 e-i and new supplementary figure 6.

3) *• Effect size: The pseudovirus and authentic SARS-CoV-2 experiments in figure 4 were performed on cells that co-express LRRC15 and ACE2-TMPRSS2. Co-expression led to very modest reductions in viral infection (~2-fold pseudovirus luciferase reduction, ~10% difference in cell survival with significant results using only 1 out of 2 authentic SARS-CoV-2 strains and only at a single MOI). Experiments detailing if LRRC15makes a significant reduction in the log10 titer of secreted infectious authentic SARS-CoV-2 would help to clarify the relative effect size.*

**Response:** We have addressed this concern in multiple ways. We see significant reduction in pseudovirus infection across a log dose-response (see new supplementary Figure 4 C and D). We also redid the authentic SARS-CoV-2 infections where we evaluate the ability of LRRC15 expressed in cis to protect these cells from cell death. We see mild protection across a log dose response, but this did not reach significance, thus we conclude that LRRC15 does not protect cells in cis. This new data is presented in supplementary figure 4E.

*4) However, as the authors note LRRC15 and ACE2 expression are mutually exclusive in the lung and thus this is more a proof of concept than a physiologic model of infection. To produce a more physiologically relevant system the authors perform a single co-culture experiment with pseudovirus but not authentic SARS-CoV-2.*

**Response:** We have now added experiments with authentic SARS-CoV-2. We infect SARS-CoV-2 permissive cells in the presence of control fibroblasts, or fibroblasts expressing LRRC15. We then collect supernatants, and perform a bioassay on new SARS-CoV-2 HEK293T-ACE2-TMPRSS2 permissive target cells to assess the degree by which LRRC15 can suppress productive infection. We found that LRRC15 expression could significantly antagonize productive SARS-CoV-2 infection across doses, however the effect is modest, and we acknowledge this in the results section. Please see new Figure 5h.

*5) It is also not clear why a 2:1 ratio of fibroblasts to target cells were used or why the cells and virus were incubated in suspension prior to seeding on a plate. Are lung luminal epithelial exposed LRRC15 fibroblasts present in a 2:1 ratio compared to SARS-CoV-2 target cells in the lung? Why was the incubation done in suspension?*

*Doesn’t a 2D co-culture more closely mimic what would be present on a lung epithelial surface rather than free floating fibroblasts? Additionally, to understand the importance of a trans suppression mechanism, these studies should be done using authentic SARS-CoV-2.*

**Response:** We apologize for leaving this information out. By single cell seq analysis, we see that the fibroblast to epithelium ration in the COVID infected lung is 2:1. We have now included this information in new figure 5f. We initially did the co-culture experiments with suspension cells to try and increase cell/virus interactions, however we have re-done the coculture experiments using adherent cells and both pseudovirus and authentic SARS-CoV-2, and we still see a modest but significant protective effect. Please see new Figure 5g and h.

*6) • Relative positioning of LRRC15 fibroblasts to target cells: In physiologic states lung fibroblasts are positioned subepithelial but in pathologic states, such as viral infection, epithelial cell death can lead to exposure of some fibroblasts. Is it known from human or mouse studies at what time post-infection fibroblasts are exposed on the luminal surface of SARS-CoV-2 infected lungs and to what degree? This is an important point, as LRRC15 expressing fibroblasts would need to be exposed to the luminal surface in sufficient numbers to sequester virus. Given what appears to be a small effect size, the number/ratio of fibroblasts would need to be significant.*

**Response:** We agree with the reviewer this is an important issue. We cite a recent spatial transcriptomics study of human COVID-19 lungs (<https://doi.org/10.1038/s41586-021-03475-6>) that found fibroblasts interact closely with Spike+ macrophages and DCs, although from this study it is not clear if these fibroblasts are in the airway. Moreover, a recent spatial transcriptomics study on bioarchives (<https://doi.org/10.1101/2021.11.26.470108>) shows fibroblasts can be found in the airways (e.g. figure 2E), however this tissue is not from COVID-19 patients and LRRC15+ fibroblasts are not discussed. Since submission we have added new histology data from human COVID-19 infected lungs, which show intense LRRC15 protein in the airways of COVID-19 patient lungs but not control lungs. Importantly we see that in COVID-19 lungs, LRRC15 protein actually lines the airways, and is mutually exclusive with collagen. Please see new figure 4 e-i and new supplementary figure 6. We hope this addresses the reviewer's concern.

***7) • Timing post-infection.*** *The presence of LRRC15 fibroblasts in COVID-19 in Figure 5 is based on scRNA-seq data obtained from rapid autopsy (Melms et al, Delorey et al.) or lung transplant (Buechler et al.). In each of these studies, these tissues are obtained at relatively long time points post-infection/symptom onset (median 27.5 days Melms, 15 days Delorey, Months Buechler, time post-symptom onset). It is known and demonstrated in these papers that viral loads are rapidly decreasing relatively early in disease (see figure 3 Delorey for example) and fibrosis increasing later in disease (see fig 4 Melms for example). Since the majority of these samples were taken from time points when virus was undetectable or present predominantly in myeloid cells (that do not express known entry receptors) it is difficult to know if sufficient numbers of LRRC15 pathologic myofibroblasts are present during early infection when virus is circulating to play an important/non-trivial role in sequestering SARS-CoV-2.*

*For these reasons, the data does not show that LRRC15 acts through a sequestration mechanism to play a significant role as a regulator of SARS-CoV-2 infection.*

**Response**: We appreciate the point this reviewer is making. We have evaluated LRRC15 protein expression in human lungs, where we see LRRC15 lining the airways in COVID-19 patients, please see new figure 4 e-i and new supplementary figure 6. The reviewer is correct, though, the data we provide is from end stage COVID-19 and not early in infection, as there is much less of this data available since tissue biopsies would be difficult to justify taking from patients that may soon become critically ill. There is one longitudinal study from primates (<https://doi.org/10.1038/s41467-022-28315-7>), but this was based on BAL samples which do not contain many fibroblasts. Importantly, a recent longitudinal study looking at serum from human COVID-19 patients found the number one most important serum protein for predicting COVID-19 severity was LRRC15, with lower LRRC15 levels predicting worse outcome (please see <https://doi.org/10.1101/2022.04.29.22274267>). While these data are highly supportive of our work, overall we agree with the reviewer that we have not definitively proven that LRRC15 expressed on fibroblasts in human lung patients is protecting these patients from severe COVID-19 at early stages of infection. As such, we have toned down our claims.

*8) In order to support the very strong claim that LRRC15 is a MASTER regulator of SARS-CoV-2 infection, an LRRC15 knockout animal model infected with SARS-CoV-2 would be required.*

**Response**: We agree that an LRRC15 KO mouse will be an important tool for us to better understand LRRC15 function *in vivo*, although this is assuming the human and mouse immune systems use the same mechanisms, which is not always the case. The patient data that shows LRRC15 serum levels is the most important predictor of COVID-19 outcome is, we think, sufficient to establish the in vivo relevance of this new receptor in human COVID-19 immunity (please see <https://doi.org/10.1101/2022.04.29.22274267>). We agree that defining the mechanisms involved in this effect would be much easier to assess in KO mice (if the mouse immune system works the same). We are now making an LRRC15 KO mouse, but this will take years to phenotype and we believe this is beyond the scope of this current study. We have toned down our claims on the importance of LRRC15 in COVID-19 infection, and we hope our updated manuscript is an acceptable compromise.

*9. It has been described that LRRC15 is a marker of myofibroblasts, LRRC15 is upregulated by cytokines, and that pathologic fibroblast are increased in the fibrotic phase of severe COVID-19 patients. Thus, the data in figure 6 suggesting that LRRC15 expression regulates collagen production is intriguing. However, the authors present data related to a single lo and hi transfection study that suggests that exogenous overexpression of LRRC15 can modulate COL1A1 expression.*

*Without mechanistic insight into how LRRC15 regulates COL1A1 or data showing this regulation occurs under endogenous expression conditions of LRRC15 it is hard to understand the importance of this finding.*

**Response**: Our initial work on LRRC15 low and high cells was with transient transfection, which can impact cell responses. To provide more insight into how LRRC15 is working in a resting cell, we created stable fibroblast cell lines expressing either GFP or LRRC15. We then performed RNA seq on these cells, and found that LRRC15 drives a clear IFN-like antiviral transcriptional response, while suppressing collagen expression. These data are now included in new figure 6 b-f. While we appreciate that more mechanistic insight into LRRC15 function would be optimal, we have not yet been able to figure out at the molecular level how LRRC15 can control transcriptional programs in fibroblasts, and since this molecule is relatively uncharacterized, we believe this will take us years and is beyond the scope of the current study. We hope the reviewer can appreciate this.

*10) Line 23: the statement that “systematic assessment of factors controlling SARS-CoV-2 host interactions has not been described” is not clear. Other systematic screens have been done but with different readouts that discovered other factors important to SARS-CoV-2. Would modify to be more specific about what is different about this screen.*

**Response**: We have rephrased this statement to say “ a systematic assessment of host factors that regulate binding to SARS-CoV-2 spike protein has not been described.”

*11) Line 189: As described above, although LRRC15 cells were not infected in these autopsy studies, viral infection was low if not absent in the vast majority of cells, including known target cells.*

**Response**: We have added text to the manuscript noting this, stating “Of note, the viral RNA detected was low in these patients.”

*12) Line 669 and 677: The “immobilization” assay referred to in the methods section is called a Pseudolentivirus sequestering assay in Figure 5G and as co-culture in the text. It would be less confusing to establish a single name for this assay.*

**Response**: We have changed this and now refer to this assay as “co-culture” throughout the manuscript.

*13) Throughout text: Authors refer to “live” SARS-CoV-2. Many would argue that viruses are not alive. Consider using authentic or infectious in lieu of “live”.*

**Response**: We have made the suggested change.
